# Library siRNA-generating RNA nanosponges for gene silencing by complementary rolling circle transcription

**DOI:** 10.1038/s41598-017-10219-y

**Published:** 2017-08-30

**Authors:** Sangwoo Han, Hyejin Kim, Jong Bum Lee

**Affiliations:** Department of Chemical Engineering, University of Seoul, 163 Seoulsiripdaero, Dongdaemungu, Seoul 02504 Republic of Korea

## Abstract

Since the discovery of RNA interference (RNAi), small interfering RNA (siRNA) has been powerful tools for gene downregulation in biomedical applications. Despite the outstanding efficacy of siRNA, the development of a therapeutic delivery system remains a challenge owing to the instability of RNA. In this study, we describe a new method for the design of siRNA-generating nanosponges by using complementary rolling circle transcription (cRCT), a technique that requires two complementary circular DNA. The sequences of one of the circular DNA are designed to have complete complementarity to the target mRNA resulting in double stranded RNA (dsRNA) that can be digested to siRNA by cellular Dicer activity. This siRNA design, called ‘library siRNA’, could be universally applied to fabricate RNA nanosponges targeting any known mRNA sequence.

## Introduction

**Figure 1:**
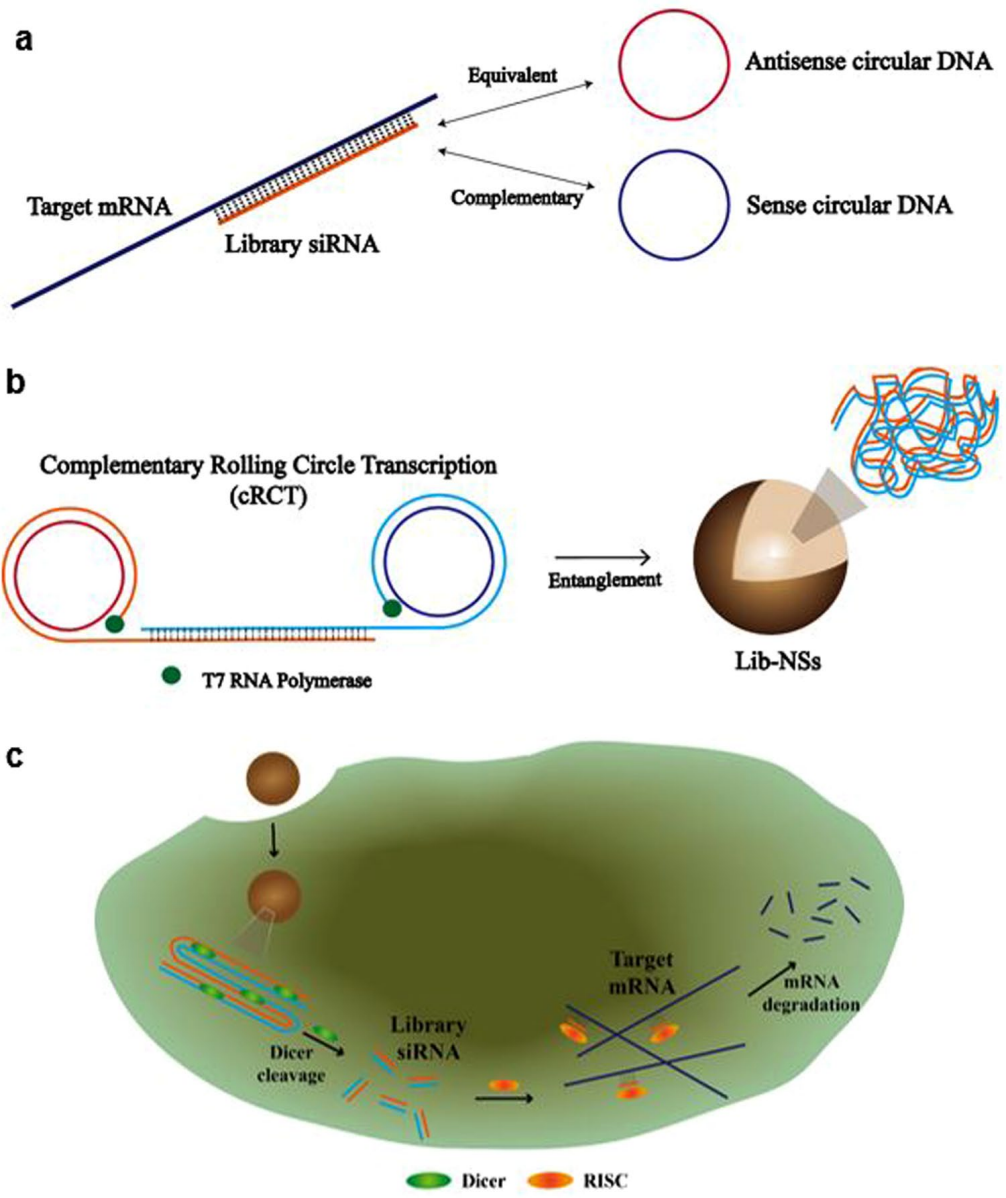
Schematic illustration of GFP lib-NSs synthesis. (**a**) Preparation of sense and antisense circular DNA templates from a target mRNA sequence. (**b**) The process of complementary rolling circle transcription (cRCT). Two RNA strands hybridize and become entangled, producing lib-NSs. (**c**) Lib-NSs enter tumour cells by endocytosis and are cleaved by Dicer (green ellipses), producing library siRNA, which complexes with RISC (orange ellipses) and target mRNA, resulting in mRNA degradation.

The development of RNA-based therapeutic treatments has been hindered by structural complexities such as intra-strand double helices, diverse tertiary and quaternary structures, and instability caused by the extra hydroxyl group in the RNA pentose ring^1^. However, owing to its similarities to DNA, RNA nanotechnology has been developed using numerous established DNA engineering techniques^2, 3^. For instance, simple hybridization for constructing DNA lattices using sticky ends^4^, enzymatic replication such as rolling circle amplification (RCA)^5–8^, and DNA origami^9^ have been in use for decades. There have been many attempts to construct RNA nanoarchitectures reflecting the myriad of biological functions of RNA^10–12^. One of the most studied types of functional RNA is small interfering RNA (siRNA), a powerful tool for regulating target gene translation^13^. Various strategies have been devised for efficient, stable siRNA delivery^14–17^. Rolling circle transcription (RCT) is an RNA polymerase-based enzymatic replication method that has been extensively used to construct siRNA-containing nanoparticles^18–25^. Sponge-like RNA nanoparticle and microspheres recently produced by RCT, which can contain over 500,000 repeated siRNA units, have much higher stability against degradation caused by serum and achieve more efficient cellular uptake than in previous studies^20^. Using this RCT technique, we suggest that siRNA-generating nanosponges can be universally designed and fabricated through complementary rolling circle transcription (cRCT)^24^, which uses two complementary circular DNA templates to produce two long RNA strands. One circular DNA template contains the equivalent target mRNA sequence (antisense circular DNA) and the other contains a completely complementary sequence to the first circular template (sense circular DNA) (Fig. 1). After transcription, the resultant RNA strands hybridize to form double stranded RNA (dsRNA) and become entangled, forming a large mass of RNA from which mRNA-targeted siRNA, called ‘library siRNA’, can be fabricated. As the RNA nanosponges are completely composed of dsRNA, there are numerous sites for cleavage by cytoplasmic Dicer, resulting in mRNA degradation. In this study, green fluorescent protein (GFP) mRNA was chosen as a target, and a GFP library siRNA nanosponges (GFP lib-NSs) was synthesized and tested for gene silencing effects. This approach could be universally used to manipulate gene expression, since library siRNA sequences can be easily designed from target mRNA.

## Results and Discussion

### Synthesis of self-assembly of library siRNA nanosponges

As a novel gene regulating system, we prepared two different circular DNAs for the synthesis of siRNA-generating nanosponges (Fig. 1a). Circular DNA was prepared as previously reported^9^. The antisense circular DNA was designed to contain a 70-bp sequence corresponding to the target mRNA, and the sense circular DNA is designed to contain the complementary sequence. The circular DNA also contained T7 promoter regions for T7 RNA polymerase binding. Table 1 shows the two linear DNA sequences, which are complementary, except for the T7 promoter binding regions. The complementary sequences enable the production of dsRNA that can be cleaved by Dicer to form a library of siRNA nanosponges (lib-NSs). After the preparation of circular DNA, as depicted in Fig. 1b, two complementary circular DNA templates were elongated by cRCT with T7 RNA polymerase. The resultant RNA strands hybridize to form dsRNA and become entangled, forming lib-NSs. By controlling the concentrations of circular DNA and T7 RNA polymerase, it is possible to adjust the size of the lib-NSs. We could confirm that the concentration of T7 RNA polymerase affect the size of lib-NSs (Supplementary information, Fig. S6). In this fashion, we synthesized three different lib-NSs (0.3 μM/RP40, 2.5 μM/RP40, 0.5 μM/RP80). As shown in Fig. 1c, the self-assembled lib-NSs enter cancer cells by endocytosis with the help of transfection reagents, and siRNA is generated in the cytoplasm by Dicer. The siRNAs complex with the RNA-induced silencing complex (RISC), which then recognizes the complementary mRNA and triggers its degradation. In this study, GFP was targeted by lib-NSs, and green fluorescence was measured to demonstrate the efficacy of gene silencing.

**Table 1:** DNA sequences used in lib-NSs assembly.

Name	Sequence (5′ to 3′)	Modification
Sense linear DNA (92 bp)	ACG TAC GGG TGA CGA AAC GAC GTT CCA TCG CTG TTA GAC TCA GAT TGG TTG CAC TTT CAG CAC GGG TTA TTC CGA GTG AAC CGT CCA CCA TC	5′-Phosphate
Antisense linear DNA (92 bp)	ATA GTG AGT CGT ATT AAA GCA AGC TGA CCC TGA AGT TCA TCT GCA CCA CCG GCA AGC TGC CCG TGC CCT GGC CCA CCC TCG TGA TTA TCC CT	5′-Phosphate
T7 promoter (20 bp)	CCC GTA CGT GAT GGT GGA CG	

### Characterization of lib-NSs

**Figure 2:**
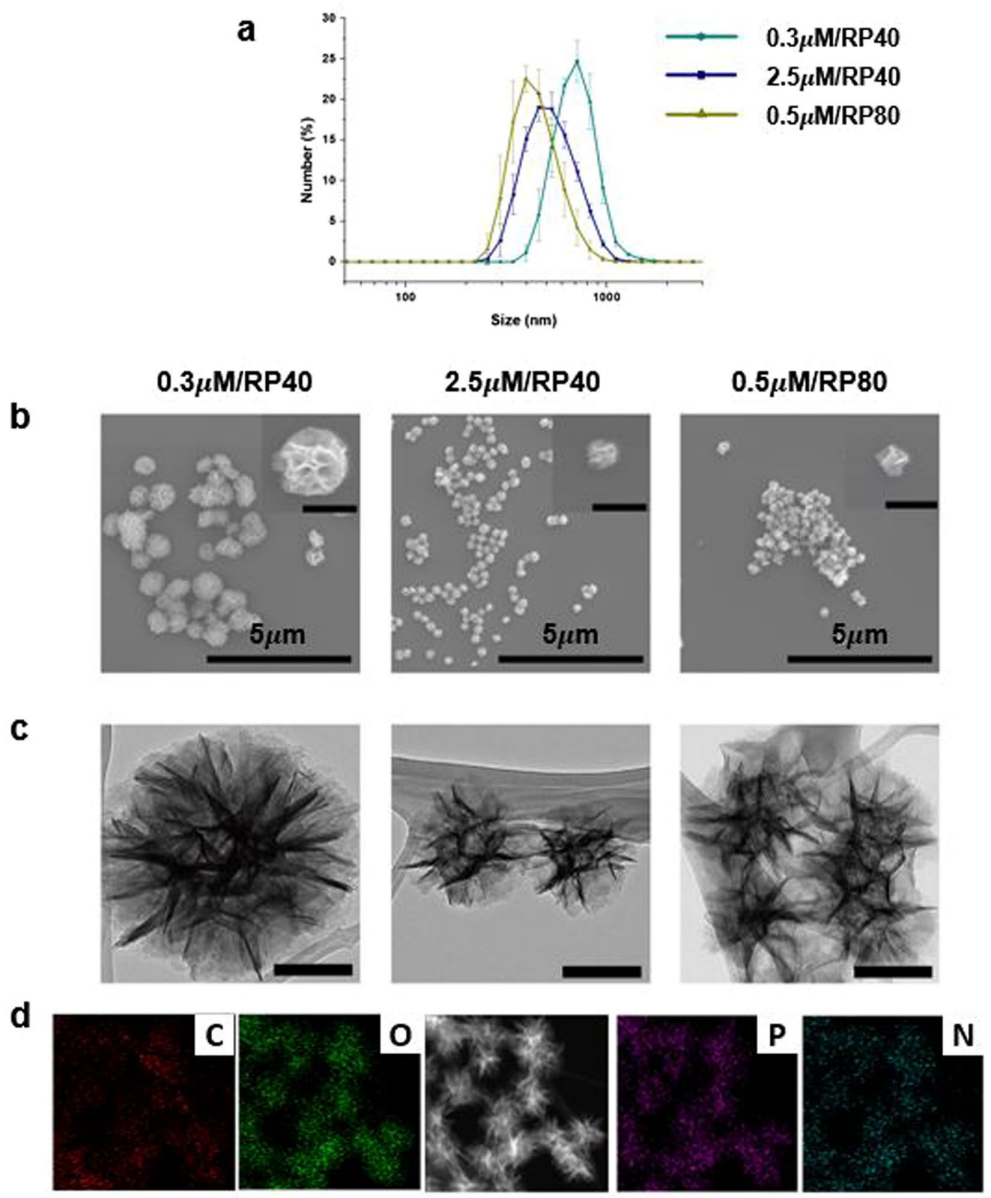
Characterization of GFP lib-NSs. (**a**) Size distribution by DLS. (**b**) SEM images. Inset: Magnified images of each GFP lib-NSs. Scale bars: 500 nm. (**c**) TEM images. Scale bars: 200 nm. (**d**) Confirmation of GFP lib-NSs components by EDS mapping.

The size, surface morphologies, and internal structures of lib-NSs were observed and measured by dynamic light scattering (DLS), scanning electron microscopy (SEM), and transmission electron microscopy (TEM). Diameters of 800, 400–500, and 300–400 nm were measured and the PDI value was 0.114, 0.123 and 0.388 for the 0.3 μM/RP40, 2.5 μM/RP40, and 0.5 μM/RP80 lib-NSs, respectively (Fig. 2a). These results were confirmed by SEM and TEM images (Fig. 2b,c). The lib-NSs were nearly spherical, and had wrinkled surface morphologies. The size of lib-NSs was well controlled by adjusting the concentration of circular DNA and T7 polymerase (Fig. 2a–c). Energy-dispersive X-ray spectroscopy (EDS) mapping analysis revealed that the particles were composed of carbon, oxygen, phosphorus, and nitrogen, consistent with RNA (Fig. 2d).

### Generation of siRNAs with recombinant Dicer

**Figure 3:**
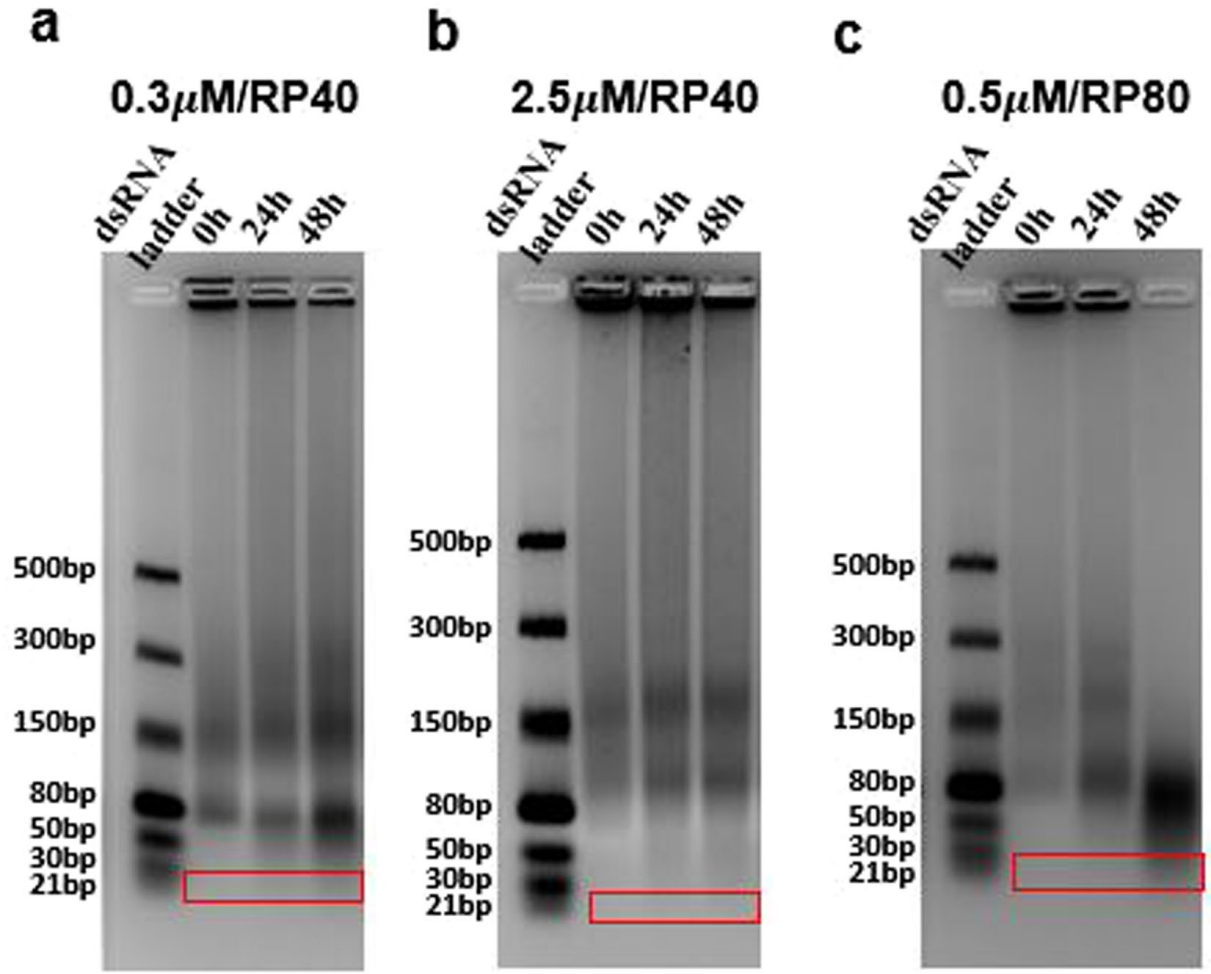
Generation of siRNA from GFP lib-NSs. Lanes 1–4 indicate the dsRNA ladder, and GFP lib-NSs 0, 24, and 48 h after Dicer treatment for 0.3 μM/RP40 (**a**), 2.5 μM/RP40 (**b**), and 0.5 μM/RP80 (**c**), respectively. dsRNA ladder and the GFP lib-NSs were loaded at the amount of 1 ${\rm{\mu }}{\rm{g}}$ in all agarose gels. The exposure was set automatically. The red boxes indicate the product generated at 21-bp region.

As illustrated in Fig. 1B, the lib-NSs are composed of numerous dsRNAs that, when cleaved by Dicer, can produce library of siRNAs. For efficient mRNA degradation in the cytoplasm, siRNAs must be generated from the nanosponges. To evaluate the length of siRNAs produced from lib-NSs of various sizes, we analyzed the products of Dicer treatment by gel electrophoresis (Fig. 3). As a result, we could confirm that the 0.5 μM/RP80 lib-NSs produced the most siRNA between 24 hours and 48 hours after Dicer treatment. Based on this result, 0.5 μM/RP80 lib-NSs were used in gene silencing assays.

### Cytotoxicity and gene silencing efficiency

**Figure 4:**
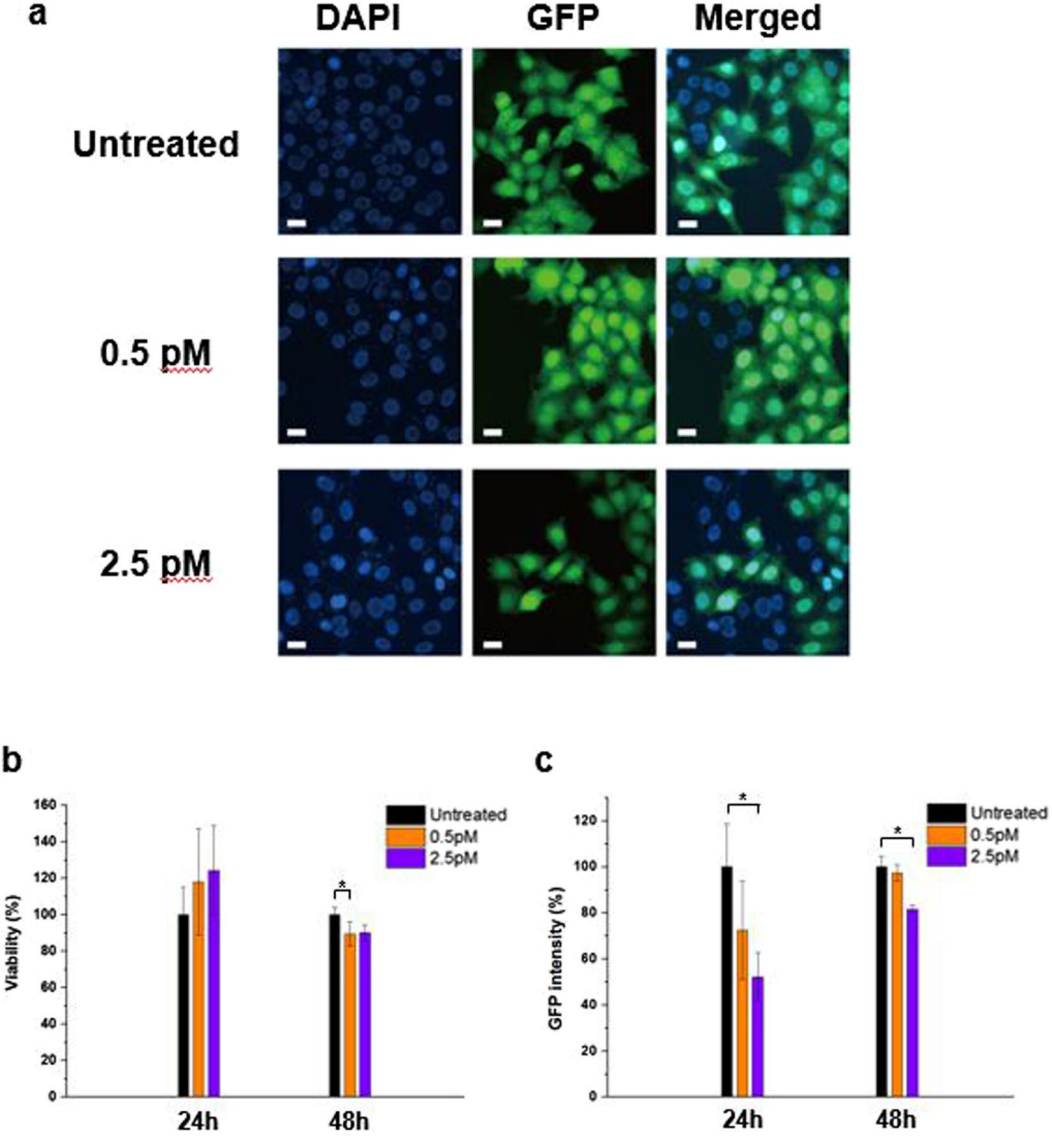
GFP lib-NSs-mediated viability and gene knockdown assays. (**a**) Fluorescence microscopy of HeLa GFP cells 24 h after treatment with 0.5 and 2.5 pM GFP lib-NSs. Control cells were left untreated for 24 h. Scale bar: 10 μm. (**b**) Viability was assayed 24 and 48 h after treatment with 0.5 and 2.5 pM GFP lib-NSs. (**c**) GFP knockdown was assayed 24 and 48 h after treatment with 0.5 and 2.5 pM of GFP lib-NSs. GFP intensities were normalized to the intensity of untreated cells (n = 4). The P-value was calculated using one-way ANOVA with a Tukey’s procedure (*p < 0.05 compared with the untreated: GFP lib-NSs).

The gene silencing efficiency of 0.5 μM/RP80 lib-NSs was tested on HeLa cells expressing enhanced GFP (HeLa GFP cells). To evaluate transfection, both poly-L-lysine (PLL) and lipid-based transfection reagents were used to deliver lib-NSs. Cytotoxicity and gene knockdown efficiency were tested (Supplementary Figs S2, S3, S5, S7). The zeta potentials of complexes prepared using 1:1 and 1:5 ratios of lib-NS and PLL were 5.43 mV and 19.1 mV, respectively (Supplementary Fig. S1). Viability assays were performed with both lib-NSs/PLL complex ratios to examine cytotoxicity (Supplementary Fig. S2). With both 1 and 4 ng/μL lib-NSs, the 1:1 ratio lib-NSs/PLL complex had low cytotoxicity; however, at the 1:5 lib-NSs/PLL complex ratio, viability decreased significantly at both lib-NSs concentrations. Lib-NSs transfected with the lipid-based transfection reagent displayed over 95% viability at 1 ng/μL, but less than 25% at 4 ng/μL GFP lib-NSs (Supplementary Fig. S2). When the lib-NSs/transfection reagent complexes had over 80% viability, a gene knockdown efficiency assay was performed (Supplementary Fig. S3). The lipid carrier complex, with viability over 90% with 1 ng/μL lib-NSs, displayed more gene knockdown capability than the lib-NSs/PLL complex (Supplementary Fig. S3), which had no effect on GFP intensity 24 h after treatment, compared to untreated HeLa GFP cells (Supplementary Fig. S4). This result suggests that PLL cannot efficiently transfect lib-NSs, either because the charge of the 1:1 lib-NSs/PLL complex is too low to transfect the lib-NSs, or because PLL could not release the lib-NSs in the endosomes. Based on these results, studies were performed to evaluate the gene regulation effects of lib-NSs. Figure 4a shows the decrease in fluorescence intensity after GFP lib-NSs treatment compared to untreated HeLa GFP cells. In addition, significant decrease of fluorescence from HeLa GFP cells was observed after 24 h incubation with 2.5 pM of lib-NSs (Fig. 4b,c). We observed approximately 50% decrease of fluorescent intensity at 2.5 pM of lib-NSs and approximately 30% decrease of fluorescent intensity at 0.5 pM of lib-NSs with stable viability (Fig. 4b,c). This result indicates that GFP lib-NSs successfully decreased GFP expression, and suggests that siRNA-generating nanosponges can be easily designed and constructed for known mRNA sequences.

## Conclusion

In conclusion, lib-NSs are a highly useful tool for constructing siRNA delivery systems by cRCT. We produced hybridized dsRNA containing complementary sequences to target mRNA from two circular DNA molecules. SEM, TEM, and DLS data suggest that the size of the lib-NSs can be controlled well by adjusting the circular DNA and T7 RNA polymerase concentrations. The generation of siRNA with recombinant Dicer allowed the evaluation of the amount of 21-bp dsRNA produced from different nanosponges, and 0.5 μM/RP80 GFP lib-NSs were demonstrated to decrease GFP expression in HeLa GFP cells without cytotoxicity. This simple method of siRNA-generating nanosponges synthesis can be readily applied to any known target mRNA.

## Materials and Method

DNA oligonucleotides were purchased from Integrated DNA Technologies (USA). T4 DNA ligase was purchased from Promega (USA). T7 RNA polymerase and 10× RNA polymerase reaction buffer were purchased from Ambion (USA). Ribonucleotide solution mix was purchased from New England Biolabs (USA). Recombinant dicer enzyme kt was purchased from Genlantis (USA). Dulbecco’s Modified Eagle’s Medium (DMEM) and fetal bovine serum (FBS) were purchased from Welgene. Antibiotic-antimycotic (100×), trypsin-EDTA, and Dulbecco’s phosphate-buffered saline (DPBS) were purchased from Gibco (USA). Stemfect RNA Transfection Reagent and Stemfect Transfection Buffer were purchased from Stemgent (USA). Cell Counting Kit-8 (CCK-8) was purchased from Dojindo (Japan).

### Circularization of linear DNA

Phosphorylated 92-bp sense and antisense linear DNA and 22-bp primer DNA, all at 10 μM, were mixed in nuclease-free water. For denaturation and annealing, the mixture was heated at 95 °C for 2 min and slowly cooled to 25 °C for 1 h in a thermal cycler (Bio-Rad). To ligate the nick in the circularized DNA, the solution was incubated overnight with 0.06 U/μL T4 DNA ligase in ligase buffer (30 mM Tris-HCl pH 7.8, 10 mM MgCl_2_, 10 mM dithiothreitol [DTT], and 1 mM ATP).

### Self-assembly of GFP lib-NSs by RCT

For three different self-assembly of GFP lib-NSs by RCT, circularized templates (at 0.3, 0.5, and 2.5 μM) were incubated with T7 RNA polymerase (at 40 and 80 U/μL) and 2 mM rNTP mix in reaction buffer (8 mM Tris-HCl, 1.2 mM MgCl_2_, 0.4 mM spermidine, and 0.2 mM DTT) for 20 h at 37 °C. One was fabricated with 0.3 μM circular DNA and 40 U/μL T7 RNA polymerase (0.3 μM/RP40), one with 2.5 μM circular DNA and 40 U/μL T7 RNA polymerase (2.5 μM/RP40), and one with 0.5 μM circular DNA and 80 U/μL T7 RNA polymerase (0.5 μM/RP80). The solution was centrifuged at 6,800 × g for 5 min to remove the supernatant, and nuclease free water was added to wash the particles. This washing step was repeated twice more to remove the RCT reagents. To break up potential aggregates, the final reactant was sonicated for 5 min.

### Characterization of GFP lib-NSs

The size of the GFP lib-NSs was measured using a Malvern Zetasizer Nano-ZS90 and analysed with Zetasizer software (Malvern Instruments). The samples were prepared immediately before use and diluted in nuclease free water. Measurements were carried out at 25 °C, and three measurements with at least 11 sub-runs were performed. GFP lib-NSs morphologies were confirmed using a Hitachi S-4300 field emission SEM. The samples were dropped and dried on silicon wafers and coated with Pt for SEM. The internal structures of GFP-lib NSs were confirmed using a JEOL JEM-2100F TEM. The samples were dropped and dried on a grid.

### siRNA generation from GFP lib-NSs

GFP lib-NSs were incubated with 0.08 U/μL of recombinant human Dicer enzyme (Genlantis) in reaction buffer (0.42 mM ATP, 1.04 mM MgCl_2_) at 37 °C for 24 and 48 h. Before the gel electrophoresis, 6X gel loading dye (New England BioLabs) and GelRed (Biotium) were mixed with each sample. The cleaved products were examined by 3% agarose gel electrophoresis at 90 V for 100 min after staining with GelRed.

### Cell culture

HeLa GFP cells (kindly provided by the Korea Institute of Science and Technology) were grown in DMEM supplemented with 10% FBS, 100 U/mL of penicillin, 100 μg/mL of streptomycin, and 1% antibiotic-antimycotic at 37 °C in a humidified atmosphere supplemented with 5% CO_2_. The cells were passaged routinely to maintain exponential growth.

### Cell viability assay

HeLa GFP cells were trypsinised and seeded at 7,000 cells/well in black 96-well flat-bottomed plates (SPL) 24 h before transfection. The GFP lib-NSs were coated with the lipid-based transfection reagent prior to transfection based on manufacturer’s instruction for 15 min. HeLa GFP cells were incubated in DMEM with the GFP lib-NSs/transfection reagent complex for 4 h before the medium was removed and replaced with serum-free medium. After 24 and 48 h incubation, 10 ${\rm{\mu }}{\rm{l}}$ of cell counting kit-8 (CCK-8) solution was added to each well (100 ${\rm{\mu }}{\rm{l}}$/well) and the plate was incubated for 90 min. The solution was then transferred to a transparent 96-well flat-bottom plate (SPL). The absorbance was measured at 450 nm on a Synergy HT microplate reader (BioTek), and the results were analysed using Gen5 2.01 software (BioTek).

### *In vitro* gene silencing assay

After the CCK-8 containing medium for the viability test was transferred to transparent 96-well plates, the black 96-well plates were washed with DPBS for 15 min before the cells were lysed with cell lysis reagent (Sigma Aldrich). GFP fluorescence was measured at 475 and 525 nm (excitation and emission) on a Synergy HT microplate reader (BioTek) and the results were analysed using Gen5 2.01 software (BioTek).

### Imaging of HeLa GFP cells for gene silencing assay

HeLa GFP cells were trypsinised and seeded at 30,000 cells per well in 8-well flat-bottomed plates (SPL) 24 h before imaging. The medium was exchanged with fresh serum-free medium 1 h before lib-NSs treatment. The GFP lib-NSs were combined with the transfection reagent prior to transfection, according to the manufacturer’s instructions. After GFP lib-NSs transfection, HeLa GFP cells were incubated at 37 °C with 5% CO_2_ for 4 h, and then washed twice with DPBS to remove GFP lib-NSs. For imaging, the cells in the 8-well plates were fixed with 4% formaldehyde (MBiotech) and washed with DPBS. The nuclei were stained with 4′,6-diamidino-2-phenylindole (DAPI) at a final concentration of 5 μg/mL. An Eclipse Ti inverted fluorescent microscope (Nikon) was used to image the cells.

## Electronic supplementary material

Supplementary Information
