# Supplementary material for: Library siRNA-generating RNA nanosponges for gene silencing by complementary rolling circle transcription
Source: Sci Rep. 2017 Aug 30;7:10005. doi: 10.1038/s41598-017-10219-y (PMC5577100; doi:10.1038/s41598-017-10219-y)
Supplement: Supplementary file 1 — Supplementary Information [file 41598_2017_10219_MOESM1_ESM.pdf]

Supplementary Information

Library siRNA-generating RNA nanosponges for gene silencing by complementary rolling circle transcription

Sangwoo Han[†], Hyejin Kim[†] and Jong Bum Lee[†]*

[†]Department of Chemical Engineering, University of Seoul, 163 Seoulsiripdaero, Dongdaemungu, Seoul, 02504, Republic of Korea

*Fax: +82-2-6490-2364; Tel: +82-2-6490-2372; E-mail: jblee@uos.ac.kr

Supplementary methods

Zeta potential measurements of lib-NSs/PLL complex

Before the Zeta potential measurements, each different complex ratio of lib-NSs/PLL was prepared. The complex sample at 1:1 mass ratio of lib-NSs and PLL was mixed and incubated for 15 min at 25℃ and the sample was diluted in nuclease free water. The Zeta potential was measured by using Malvern Zetasizer Nano-ZS90 and anlysed with Zetasizer software (Malvern Instruments). The measurements were carried out at 25℃, and three measurements with 11 sub-runs were performed. The sample of 1:5 complex ratio was measured as same method of 1:1 complex. In case of lib-NSs/lipid carrier complex, 120 ng of lib-NSs was complexed with 0.16 µl of lipid-based transfection reagent for 15 min at 25℃ and the Zeta potential was measured as same method with lib-NSs/PLL complex.

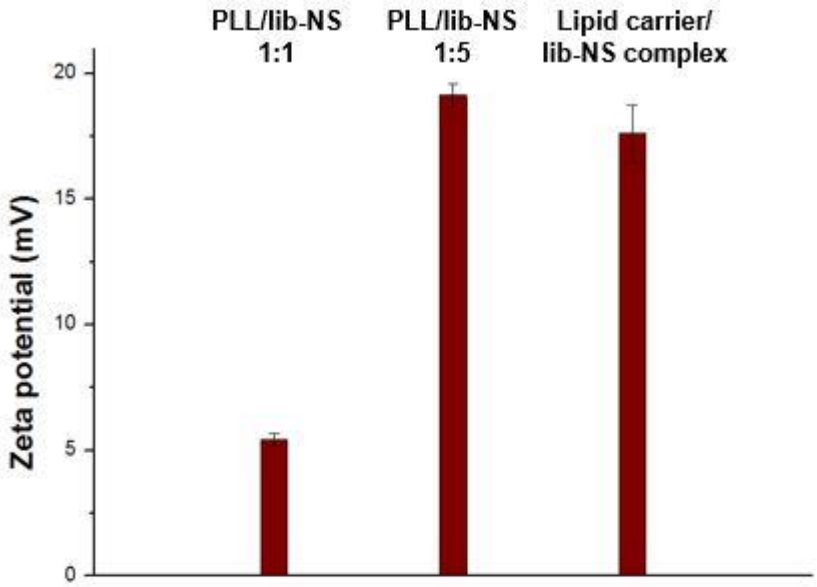

Figure S1. Zeta potential of PLL/lib-NSs and lipid carrier/lib-NSs complex. Zeta potential of 1 : 1 complex is 5.43mV, 19.1mV at 1 : 5 complex and 17.6mV at lipid carrier complex. Each complex was measured three times.

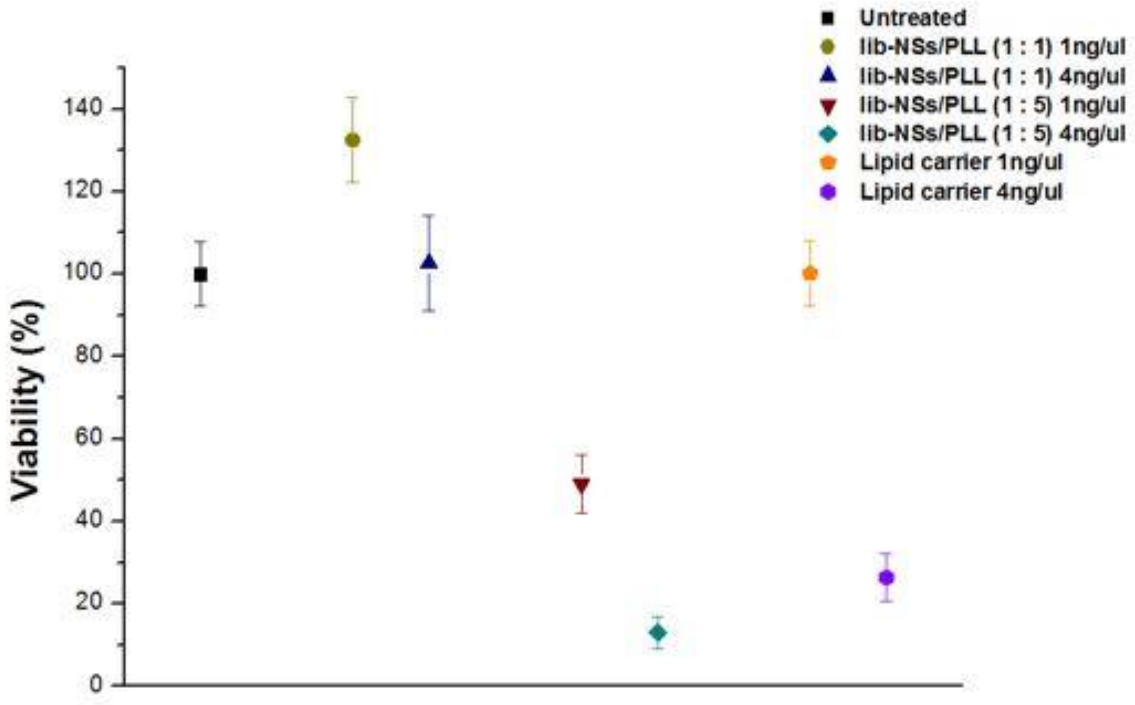

Figure S2. Viability assay. Viability assay was analyzed at 24 hours after the lib-NSs/PLL complex and lib-NSs/lipid carrier complex treatment.

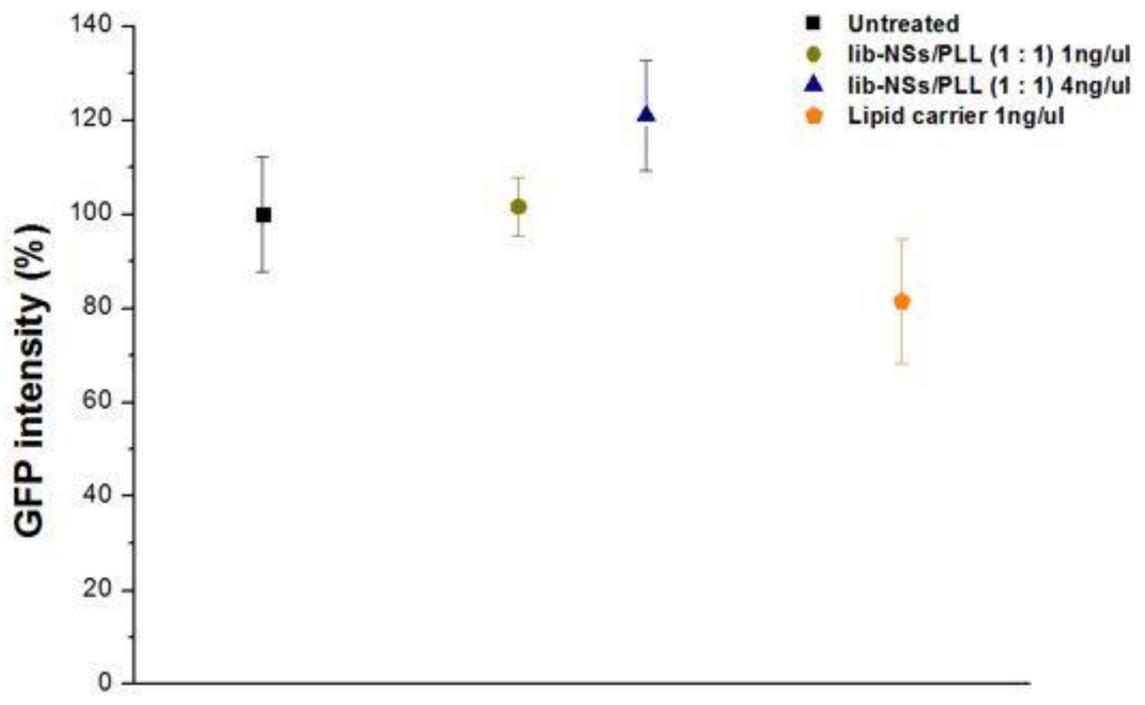

Figure S3. Gene silencing effect assay. GFP intensity was measured at 24 hours after the lib-NSs/PLL complex and lib-NSs/lipid carrier complex treatment.

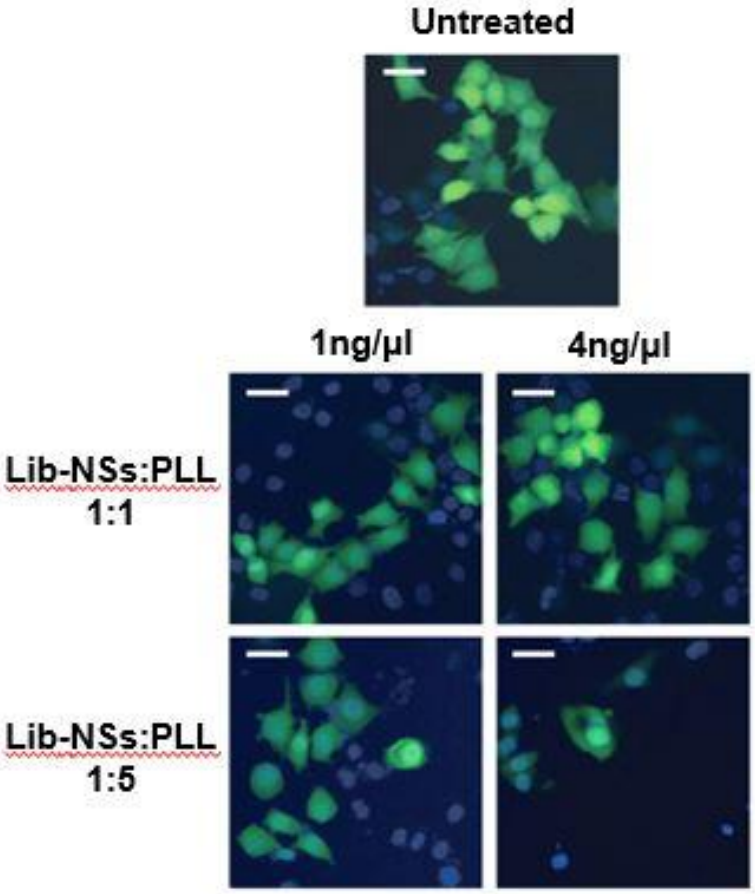

Figure S4. Fluorescence microscopy images for gene knockdown. GFP expression is observed by fluorescence microscopy. The decrease of GFP intensity was not observed at all concentration. Living cells were not able to be observed when 4 ng μl^{-1} of 1:5 lib-NSs/PLL complex was treated on Hela GFP cells. Scale bar: 20 μm.

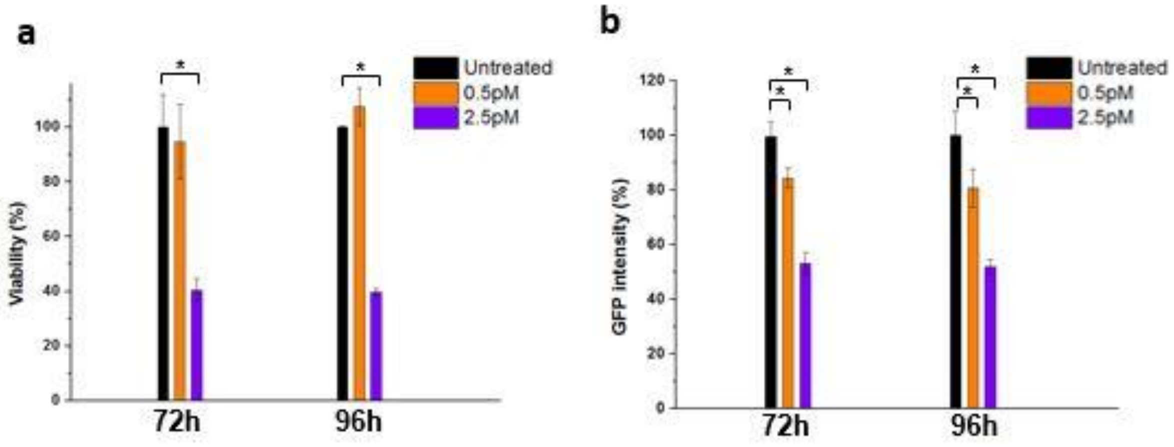

Figure S5. GFP lib-NS-mediated viability and gene knockdown assays after 72 hours and 96 hours. (a) Viability was assayed 72 and 96 h after treatment with 0.5 and 2.5 pM of GFP lib-NSs. (b) GFP knockdown was assayed 72 and 96 h after treatment with 0.5 and 2.5 pM of GFP lib-NSs. GFP intensities were normalized to the intensity of untreated cells (n=4). The P-value was calculated using one-way ANOVA with a Tukey's procedure (* $p < 0.05$ compared with the untreated:GFP lib-NS).

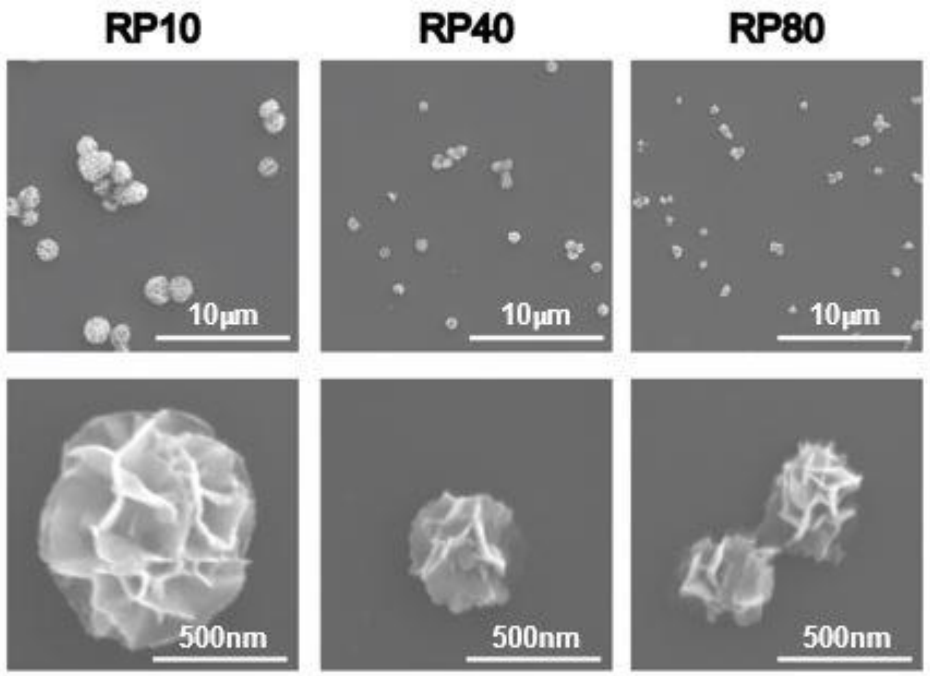

Figure S6. Size control by adjusting the concentration of T7 RNA polymerase. The diameter is approximately 1µm at RP10, 500nm at RP40 and 300nm at RP80.

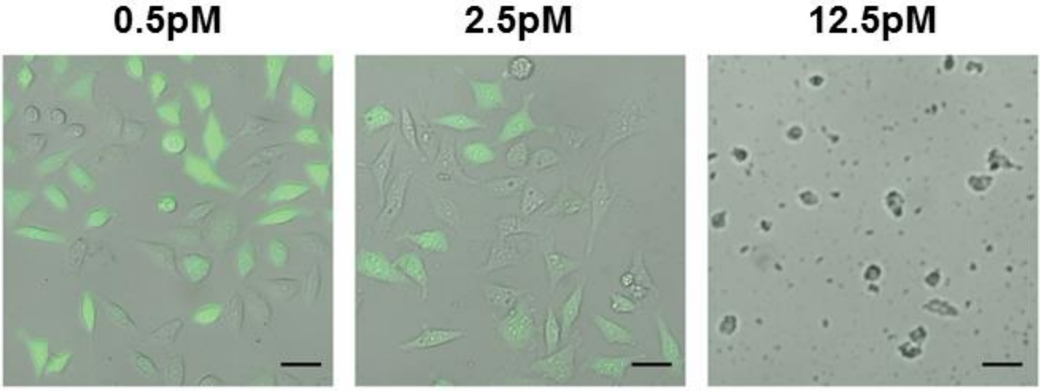

Figure S7. Viability and GFP downregulation assays. At 12.5 pM of GFP lib-NSs treatment, the viability of HeLa GFP cells was dramatically decreased compared to 0.5 pM and 2.5 pM concentration. Scale bar: 20 μm.
